# Oncogenic features of neuromedin U in breast cancer are associated with NMUR2 expression involving crosstalk with members of the WNT signaling pathway

**DOI:** 10.18632/oncotarget.16121

**Published:** 2017-03-11

**Authors:** Stefan Garczyk, Natalie Klotz, Sabrina Szczepanski, Bernd Denecke, Wiebke Antonopoulos, Saskia von Stillfried, Ruth Knüchel, Michael Rose, Edgar Dahl

**Affiliations:** ^1^ Molecular Oncology Group, Institute of Pathology, Medical Faculty of the RWTH Aachen University, D-52074 Aachen, Germany; ^2^ IZKF Aachen, Medical Faculty of the RWTH Aachen University, D-52074 Aachen, Germany

**Keywords:** breast cancer, metastasis, neuromedin U, neuromedin U receptor 2, WNT signaling

## Abstract

Neuromedin U (NMU) has been shown driving the progression of various tumor entities, including breast cancer. However, the expression pattern of NMU and its receptors in breast cancer tissues as well as systematic insight into mechanisms and downstream targets of the NMU-driven signaling pathways are still elusive. Here, NMU expression was found up-regulated in all breast cancer subtypes when compared to healthy breast tissue. Using an *in silico* dataset comprising 1,195 samples, high *NMU* expression was identified as an indicator of poor outcome in breast tumors showing strong *NMUR2* expression. Next, the biological impact of NMU on breast cancer cells in relation to NMUR2 expression was analyzed. Ectopic NMU expression reduced colony growth while promoting a motile phenotype in NMUR2-positive SKBR3 but not NMUR2-negative Hs578T cells. To uncover signaling pathways and key molecules affected by NMU in SKBR3 cells, Affymetrix microarray analysis was applied. Forced NMU expression affected molecules involved in WNT receptor signaling among others. As such we demonstrated enhanced activation of the WNT/planar cell polarity (PCP) effector RAC1 and down-regulation of canonical WNT targets such as MYC. In summary, NMU might contribute to progression of NMUR2-positive breast cancer representing a potential druggable target for future personalized strategies.

## INTRODUCTION

Breast cancer is the most frequently diagnosed and the second most common cause of cancer deaths in females in industrialized countries [1]. Breast cancer-related death is mainly due to metastasis. Deciphering novel molecules of key signaling pathways orchestrating cancer cell dissemination from the primary tumor and migration and invasion into the surrounding stroma is therefore desirable [2].

The neuromedin peptide family members were initially discovered in porcine spinal cord isolates, characterized by their ability to stimulate smooth muscle contraction but have also been reported being involved in pathophysiological processes like inflammation and carcinogenesis [3]. The name neuromedin U (NMU) originates from its observed contractile effects on the uterus of rats [4]. Human prepro-NMU, a 19 kDa protein composed of 174 amino acids, is processed into the active peptide NMU-25, consisting of 25 amino acid residues [3]. NMU is expressed in various organs and tissues showing the strongest expression in the gastrointestinal tract and distinct regions of the brain including the pituitary gland [5–7]. The numerous physiological effects attributed to NMU are mediated by at least two G-protein-coupled receptors, namely neuromedin U receptor 1 and 2 (NMUR1 and 2) whose expression patterns overlap that of NMU. NMUR1 is predominantly expressed in peripheral tissues, especially the gastrointestinal tract [7], whereas NMUR2 expression is pronounced in the central nervous system [8]. Besides effects on smooth muscle contraction [9], NMU has been demonstrated to have anorexic activity thereby regulating feeding behavior and energy homeostasis [10, 11]. Moreover, NMU has been associated with vasoconstriction [12], nociception [13], circadian control [14] and bone formation [15]. In carcinogenesis of various tumor entities, current data suggest an oncogenic role for NMU mainly in disease progression, probably by promoting cell motility and the invasive capacity of cancer cells [16–21]. In breast cancer, Rani et al. described NMU for the first time as a candidate drug response biomarker for HER2-targeted therapies and as a putative therapeutic target to reduce metastatic spread of breast cancer cells [20]. Nevertheless, the expression pattern of NMU and its potential receptors in breast cancer tissues remains elusive and a systematic analysis of signaling pathways and associated molecules affected by NMU in breast cancer is still missing.

In the present study, we demonstrate up-regulation of NMU expression in breast cancer compared to healthy breast tissues for the first time and show that high *NMU* mRNA expression is associated with poor outcome in breast carcinomas presenting strong NMUR2 expression. In line with this finding, we provide evidence that NMU might promote a motile phenotype of NMUR2-positive breast cancer cells. We identified for the first time a putative NMU-mediated modulation of WNT-superfamily signaling associated with enhanced activation of the small GTPase RAC1 that may contribute to increased migration of NMUR2-positive SKBR3 breast cancer cells. Therefore, we hypothesized that NMU may have an oncogenic role driving the progression of NMUR2-positive breast carcinomas potentially representing a novel target for the development of future personalized therapeutic strategies.

## RESULTS

### *NMU* mRNA expression in breast cancer and intrinsic subtypes

**Figure 1:**
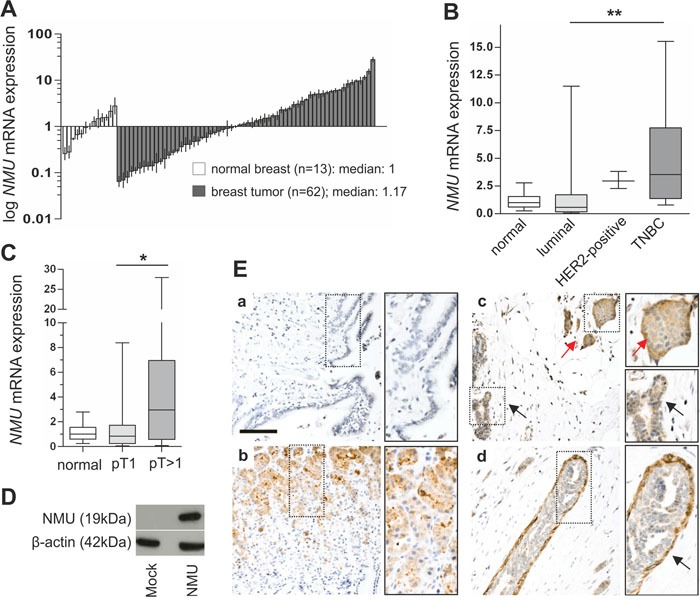
NMU expression in breast cancer and intrinsic subtypes **(A)** Real-time PCR-based *NMU* mRNA expression analysis of 62 breast tumor samples compared to 13 healthy breast tissue samples. Vertical lines: ± standard error of margin (SEM). **(B)** Box plots of the samples shown in A demonstrating a significant increase in *NMU* mRNA expression in the IHC-defined TNBC cases compared with breast carcinomas of the luminal subtype. Horizontal lines: grouped medians. Boxes: 25-75% quartiles. Vertical lines: range, minimum and maximum, TNBC: triple negative breast cancer, ** *P* < 0.01 (Kruskal-Wallis test). **(C)** Box plot illustrating *NMU* up-regulation in breast tumors > pT1. Horizontal lines: grouped medians. Boxes: 25-75% quartiles. Vertical lines: range, minimum and maximum, * *P* < 0.05 (Kruskal-Wallis test). **(D)** Western blot detection of full length NMU protein in Hs578T cells transfected with either NMU expression vector or empty vector control. **(E)** No staining is observed in lung tissue **(a)** serving as negative control while strong NMU protein expression is present in basal glandular cells of the stomach **(b)**. In the mammary tissue cytoplasmic NMU protein expression is enhanced in tumor cells (red arrow) compared with adjacent normal breast epithelial cells (black arrow) **(c)**. NMU protein expression is pronounced in myoepithelial cells (arrow) of the breast **(d)**. Scale bar: 100μm.

In a recent study NMU was described for the first time in human breast cancer as potential predictive biomarker for HER2-positive breast carcinomas and as a candidate therapeutic target to prevent metastatic spread [20]. However, this study lacked information about the subtype-specific expression pattern of NMU and its potential receptors in breast cancer tissues. Moreover, systematic insight into oncogenic mechanisms of NMU and modulated downstream signaling pathways in breast cancer remains elusive. Therefore we initially analyzed NMU expression in the different breast cancer subtypes. We performed a semi-quantitative *NMU* mRNA expression analysis of 62 breast cancer samples compared to 13 normal breast tissues. For cohort characteristics of analyzed samples see Supplementary File 1. Comparing all tumor samples to healthy controls, only a slight increase in *NMU* mRNA expression in tumor specimens was noted (median fold change (FC): 1.17) (Figure 1A). Classifying tumor samples by subtypes, i.e. “luminal”, “HER2-positive” and “triple-negative breast cancer (TNBC)” [22], based on immunohistochemistry (IHC) and fluorescence *in situ* hybridization (FISH) data for estrogen receptor (ER), progesterone receptor (PR) and human epidermal growth factor receptor 2 (HER2), revealed an increase in *NMU* mRNA expression in HER2-positive and triple-negative breast carcinomas (median FC: 3.0 and 3.5) (Figure 1B). The association between non-luminal breast cancer subtypes and *NMU* mRNA expression up-regulation was confirmed conducting Fisher's exact test showing a highly significant negative correlation of both a positive ER and PR status with *NMU* mRNA expression (for both P < 0.01; Table 1). Furthermore, a positive correlation of high *NMU* mRNA expression and HER2-positive cases was found (P < 0.05; Table 1). Interestingly, *NMU* mRNA expression was also significantly enhanced in advanced tumors of larger size (median FC pT1 vs. pT>1: 3.5, P < 0.05) (Figure 1C).

**Table 1:** Clinico-pathological parameters of 62 breast cancer specimens analyzed in relation to *NMU* mRNA expression

Parameter	n^a^	*NMU* high	*NMU* low	P-value^b^
Total	62	23 (33.8%)	39 (57.4%)	-
Age at diagnosis				
<63.5 years	31	11 (35.5%)	20 (64.5%)	1.000
≥63.5 years	31	12 (38.7%)	19 (61.3%)	
Tumor size^c^				
pT1	35	8 (22.9%)	27 (77.1%)	**0.016**
pT2-3	27	15 (55.6%)	12 (44.4%)	
Lymph node status^c^				
pN0	34	12 (35.3%)	22 (64.7%)	0.791
pN1-3	27	11 (40.7%)	16 (59.3%)	
Histological tumor grade^d^				
G1-2	23	6 (26.1%)	17 (73.9%)	0.275
G3	38	16 (42.1%)	22 (57.9%)	
Histological type				
invasive ductal	55	21 (38.2%)	34 (61.8%)	1.000
invasive lobular	5	2 (40.0%)	3 (60.0%)	
Estrogen receptor status				
negative (IRS^e^ 0-2)	18	12 (66.7%)	6 (33.3%)	**0.003**
positive (IRS^e^ 3-12)	42	10 (23.8%)	32 (76.2%)	
Progesterone receptor status				
negative (IRS^e^ 0-2)	20	13 (65.0%)	7 (35.0%)	**0.001**
positive (IRS^e^ 3-12)	39	8 (20.5%)	31 (79.5%)	
HER2 status^f^				
negative	53	17 (32.1%)	36 (67.9%)	**0.044**
positive	8	6 (75.0%)	2 (25.0%)	

^a^Only female patients with primary, unilateral, invasive breast cancer were included. *NMU* high expression: ≥ 2 fold change in relation to median normal breast tissue expression. ^b^Fisher's exact test. ^c^According to TNM classification by Sobin and Wittekind [52]. ^d^According to Bloom and Richardson, as modified by Elston and Ellis [53]. ^e^Immunoreactive score (IRS) according to Remmele and Stegner [54]. Significant P-values are marked in bold face. ^f^Overexpression of the *ERBB2* gene (*HER2/neu*) was diagnosed analogously to the threshold of the DAKO-Score system based on IHC assay. Uncertain cases were additionally validated by FISH assay. Percentages may not sum-up to 100% due to rounding.

Subsequently, we aimed at characterizing for the first time the NMU protein expression pattern in healthy breast and breast cancer samples using immunohistochemistry. As there is a clear lack of validated commercially available NMU antibodies, we started our analysis with a profound examination of the antibody applied in this study. The antibody's specificity was verified by performing western blot analysis of Hs578T breast cancer cells transfected with either NMU expression vector as positive or empty vector as negative control. We successfully detected the 19 kDa full-length NMU protein in the positive control whereas the signal was absent in the negative control lysate (Figure 1D). Ensuing, we validated the antibody's performance on FFPE material. As it is well known that *NMU* mRNA is highly expressed throughout the gastrointestinal system [3], human stomach served as positive control, while human adult lung was used as negative control for NMU expression [7, 17]. NMU protein was strongly expressed in basal glandular cells of the stomach (Figure 1E-1b), whereas no expression was observed in lung tissue (Figure 1E-1a). Finally, we applied the antibody to several human healthy breast and breast cancer samples. We were able to detect NMU protein only in those specimens showing very high *NMU* mRNA expression possibly due to low antibody sensitivity and/or a low half-life time of the protein [3]. In concordance with our mRNA data, we noted higher cytoplasmic NMU protein expression in cancer cells compared with adjacent normal breast epithelial cells (Figure 1E-1c). Interestingly, NMU protein expression was pronounced in the myoepithelium of mammary ducts (Figure 1E-1d), a finding in line with the well documented smooth-muscle contractile properties of NMU [23] suggesting a yet undescribed role for NMU in the contraction of mammary ducts.

### *NMU* mRNA expression is associated with advanced breast tumor stages

**Figure 2:**
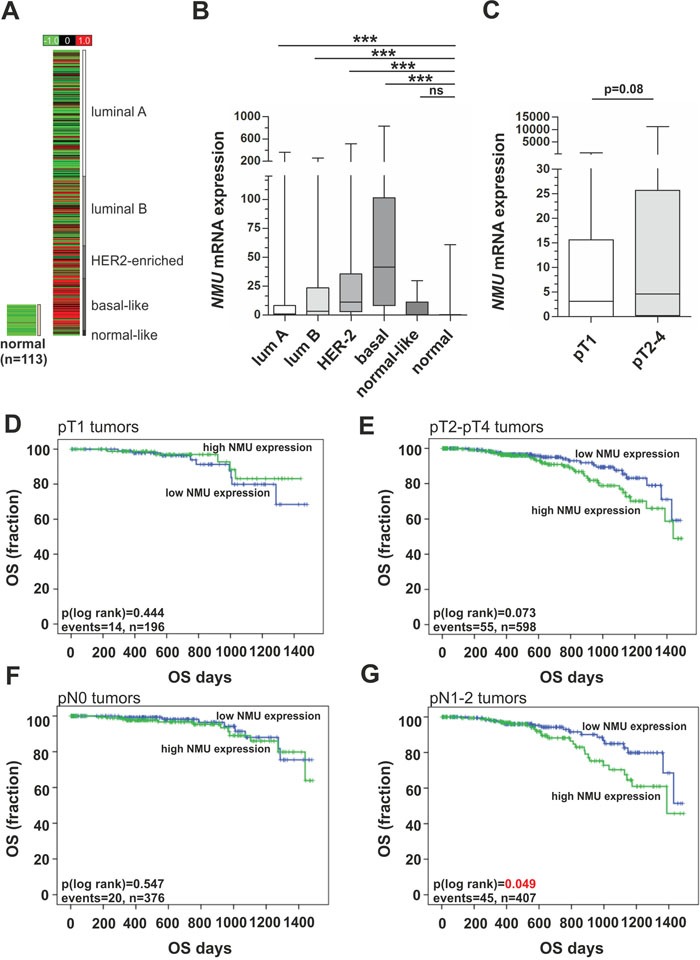
*NMU* mRNA expression in an independent breast cancer cohort and its clinical impact in advanced tumor stages **(A)** *In silico NMU* mRNA expression analysis of 830 PAM50-defined breast tumor samples and 113 normal breast tissues depicted as heat map. Red color: high, black: intermediate, green: low *NMU* mRNA expression. **(B)** Box plot demonstrating breast cancer subtype-specific *NMU* mRNA expression. Horizontal lines: grouped medians. Boxes: 25-75% quartiles. Vertical lines: range, minimum and maximum. *** *P* < 0.001; ns: not significant (Kruskal-Wallis test). **(C)** *NMU* mRNA expression is elevated in larger breast tumors. Horizontal lines: grouped medians. Boxes: 25-75% quartiles. Vertical lines: range, minimum and maximum (Mann-Whitney-U test). **(D-G)** Univariate Kaplan-Meier survival curves displaying overall survival (OS) of patients with high *NMU* (>median expression) expression (green line) in relation to low *NMU* (≤ median expression) expression (blue line) in pT1 (D) and in pT2-4 tumors (E). (F-G) Univariate Kaplan-Meier survival curves displaying overall survival (OS) of patients with high *NMU* (>median expression) expression (green line) in relation to low *NMU* (≤ median expression) expression (blue line) in pN-negative (F) and in pN-positive tumors (G).

*NMU* mRNA expression was validated in a large dataset of an independent study. Using Illumina HiSeq expression data of 830 PAM50-defined breast cancer cases and 113 normal breast tissues available at the *The Cancer Genome Atlas* (TCGA) [24], significant (P < 0.001) increase of *NMU* mRNA level in all clinically relevant breast cancer subtypes was clearly confirmed, including luminal A and B tumors when compared with normal tissues (FC mean luminal A: 6.1, FC mean luminal B: 14.4). Strongest *NMU* mRNA expression was observed in HER2-enriched (FC mean: 27.5) and basal-like breast carcinomas (FC mean: 72.4) (Figure 2A-2B). *NMU* mRNA expression was also increased in advanced breast tumors (median FC pT1 vs. pT>1: 1.46; P = 0.08; n = 1,055) (Figure 2C). By univariate Kaplan-Meier analysis we found that patients with high *NMU* expression tend (P = 0.07) to have shorter overall survival (OS) (mean OS: 1,271 days ± 32; 95% CI: 1,207 to 1,334) in the subgroup of advanced (pT2-pT4) breast tumors when compared with low NMU expression (mean OS: 1,347 days ± 45; 95% CI: 1,241 to 1,418) (Figure 2D-2E). No significant association between NMU expression and OS was noted in the subgroup of small (pT1) breast carcinomas. Interestingly, a significant correlation between NMU expression and OS was also observed in nodal-positive (pN1-2) patients (Figure 2G) whereas no significant association in patients with a nodal-negative (pN0) status was noticed (Figure 2F). These findings support the hypothesis that NMU may play a critical role in breast cancer progression.

### Clinical impact of NMU ligand in dependency of NMU receptor expression

**Figure 3:**
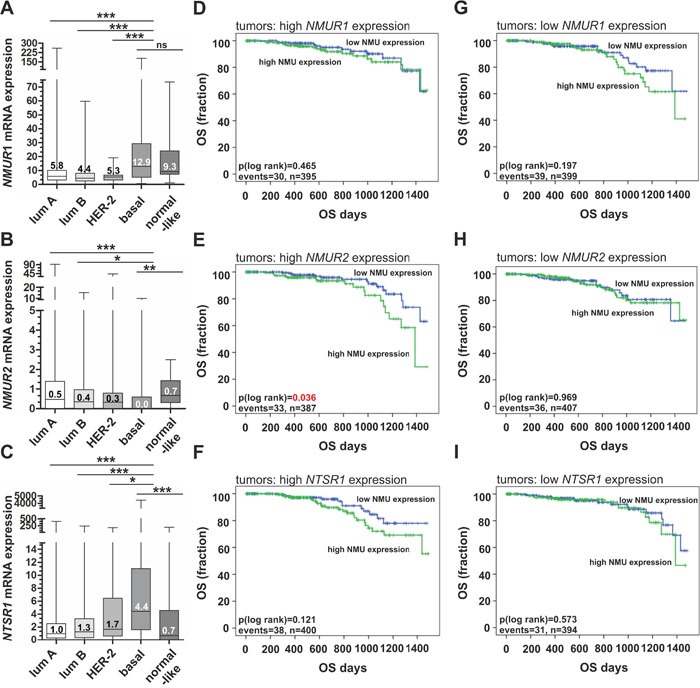
Expression of NMU receptors in breast cancer subtypes and their clinical impact in dependency of NMU ligand expression **(A-C)** Box plots showing breast cancer subtype-specific *NMUR1* (A), *NMUR2* (B) and *NTSR1* (C) mRNA expression. Horizontal lines: grouped medians. Boxes: 25-75% quartiles. Vertical lines: range, minimum and maximum. * *P* < 0.05, ** *P* < 0.01, *** *P* < 0.001; ns: not significant (Kruskal-Wallis test). **(D-F)** Univariate Kaplan-Meier survival curves displaying overall survival (OS) of patients with high *NMU* (>median expression) expression (green line) in relation to low *NMU* (≤ median expression) expression (blue line) in tumors with strong NMU receptor expression (> median expression), i.e. (D) *NMUR1* (E) *NMUR2* and (F) *NTRS1*. **(G-I)** Univariate Kaplan-Meier survival curves displaying overall survival (OS) of patients with high *NMU* (>median expression) expression (green line) in relation to low *NMU* (≤ median expression) expression (blue line) in tumors with low NMU receptor expression (≤ median expression), i.e. (G) *NMUR1* (H) *NMUR2* and (I) *NTRS1*. Vertical lines: censored cases.

The overall prognostic relevance of the ligand NMU in breast cancer subtypes has recently been reported by Rani and co-workers [20]. However, Rani et al. did not evaluate the association between NMU and patient survival in a receptor dependent manner that may help to understand the varying impact of NMU on the different breast cancer subtypes observed. In the present study we analyzed the expression of receptors which have been reported as involved in mediating NMU signaling. Based on the TCGA data set overall comprising 1,082 breast cancer samples, we demonstrated abundant *NMUR1* expression in basal-type and normal-like breast cancers. Luminal A and luminal B as well as HER2-enriched tumors were characterized by significantly lower expression levels (Figure 3A). In contrast *NMUR2* was expressed in all subtypes but basal-type carcinomas almost completely lacking *NMUR2* expression (median expression = 0) (Figure 3B). *Neurotensin Receptor 1* (*NTSR1*) mRNA was found expressed similar to *NMUR1* (Figure 3C) but *Growth Hormone Secretagogue Receptor* (*GHSR*), the correspondent receptor of the NTSR1/GHSR heterodimer receptor complex [17] was rarely expressed (median expression= 0) in all subtypes (data not shown) and thus, further statistical analysis was not valid.

Next we examined patients’ OS as indicator of a clinical impact based on the publicly available TCGA breast cancer cohort. Univariate analyses demonstrated that high *NMU* mRNA expression predicted poor prognosis (P < 0.05) only in tumors showing high *NMUR2* expression (mean OS: 1,247 days ± 44; 95% CI: 1,160 to 1,134) compared to tumors with low *NMU* expression (mean OS: 1,363 days ± 28; 95% CI: 1,306 to 1,419) (Figure 3E). No significant impact of *NMU* expression was observed in tumors with low *NMUR2* expression (Figure 3H). Furthermore, NMU expression was not significantly associated with shorter OS in any other combination, i.e. neither with *NMUR1* nor *NTSR1* expression (Figure 3D-3I). Interestingly, *NMU* tends to predict poor survival in breast tumors presenting low levels of *NMUR1* mRNA (Figure 3G). Taken together, these data suggest a potential oncogenic role of NMU especially in a NMUR2-positive background.

### NMU over-expression reduces cell growth while promoting a motile phenotype in NMUR2-positive SKBR3 breast cancer cells

**Figure 4:**
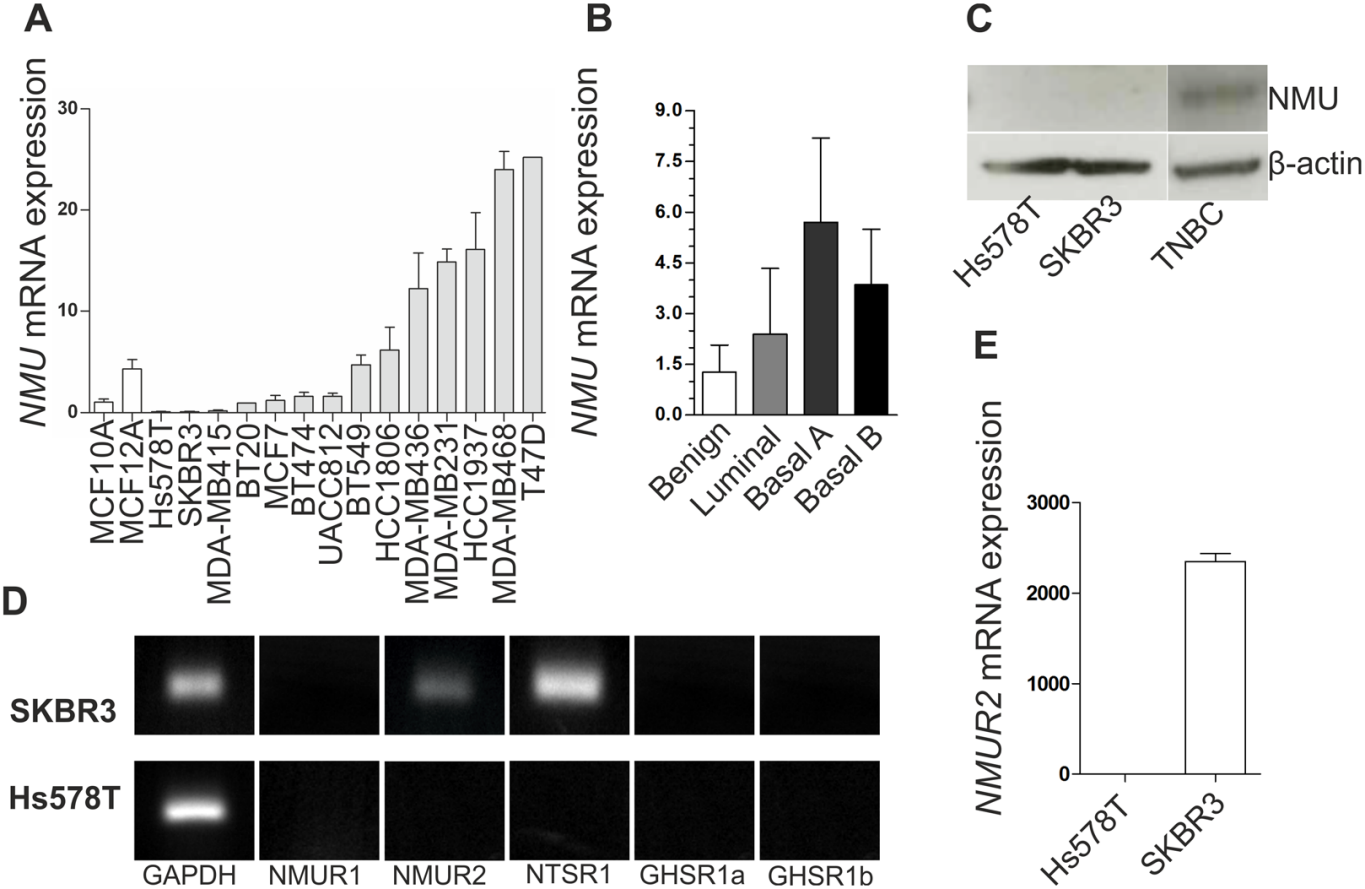
NMU ligand and NMU receptor expression in breast cancer cell lines **(A)** *NMU* mRNA expression in two benign (*white*) and 14 malignant breast cancer cell lines (*grey*) relative to the *NMU* mRNA expression in MCF10A. Vertical lines: ± standard error of margin (SEM). **(B)** *NMU* mRNA expression of the cell lines shown in A classified by molecular subtype. **(C)** NMU protein expression in the basal-like cell line Hs578T, the luminal-like, HER2-positive cell line SKBR3 and a triple negative breast carcinoma (TNBC). **(D)** mRNA expression of the NMU receptors *NMUR1* and *NMUR2*, the potential receptors *NTSR1* and *GHSR* (transcripts 1a and 1b) and *GAPDH* as loading control in SKBR3 and Hs578T cells. **(E)** Comparison of *NMUR2* mRNA expression in Hs578T and SKBR3 breast cancer cells. Vertical lines: ± 95% confidence interval (CI).

**Figure 5:**
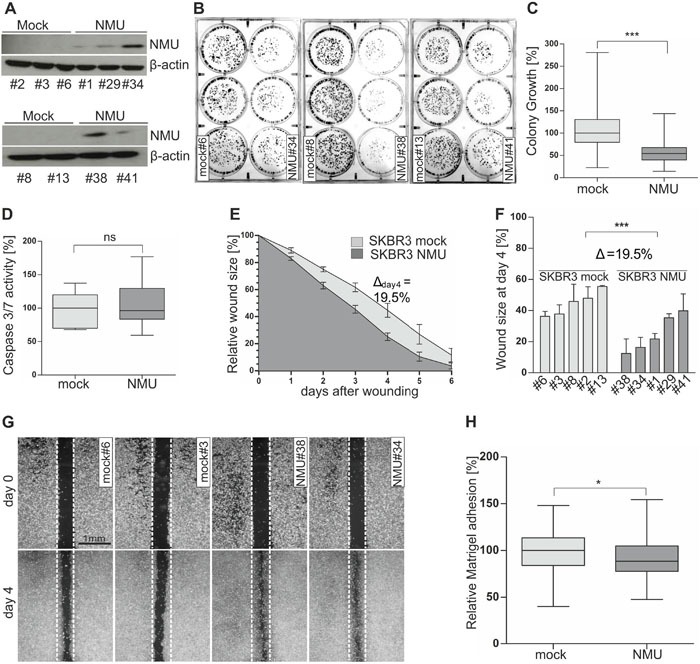
Ectopic NMU expression in NMUR2-positive SKBR3 cells reduces colony growth while promoting a motile phenotype **(A)** NMU protein expression of stably transfected independent SKBR3 mock (#2, #3, #6, #8, #13) and NMU (#1, #29, #34, #38, #41) clones. **(B)** Representative 6-well plates containing SKBR3 mock and NMU clones (two weeks after cell seeding). **(C)** Densitometrical evaluation of 2D colony growth after two weeks. Box plot presents median colony growth of triplicate experiments for SKBR3 mock (n=5) and NMU (n=5) clones. Horizontal lines: grouped medians. Boxes: 25-75% quartiles. Vertical lines: range, minimum and maximum, *** *P* < 0.001 (Mann-Whitney-U test). **(D)** Caspase 3/7 activity as indicator of apoptosis in SKBR3 mock (n=5) and NMU (n=5) clones. Box plot demonstrates median caspase 3/7 activity of three independent experiments. Horizontal lines: grouped medians. Boxes: 25-75% quartiles. Vertical lines: range, minimum and maximum, ns: not significant (Mann-Whitney-U test). **(E)** Comparison of cell migration of SKBR3 mock (n=5) and NMU (n=5) clones analyzed by monolayer wound healing assay over 6 days. Vertical lines: standard deviation of triplicates. Cell-free area on day 0 was set 100% and used for standardization. Δ_day4_: differences of cell-free areas on day 4. **(F)** Detailed comparison of wound closure for each clone after 4 days, vertical lines: standard deviation of triplicates, *** *P* < 0.001 (Mann-Whitney-U test), Δ: difference of cell-free areas on day 4. **(G)** Wound area documentation by phase contrast microscopy at time point 0 and 4 days after start of experiment. Scale bar: 1000μm. **(H)** Cell-matrix adhesion of stably transfected SKBR3 mock (n=5) and NMU (n=5) clones was measured colorimetrically. Box plot shows median values of 5 independent experiments. Horizontal lines: grouped medians. Boxes: 25-75% quartiles. Vertical lines: range, minimum and maximum, * *P* < 0.05 (Mann-Whitney-U test).

**Figure 6:**
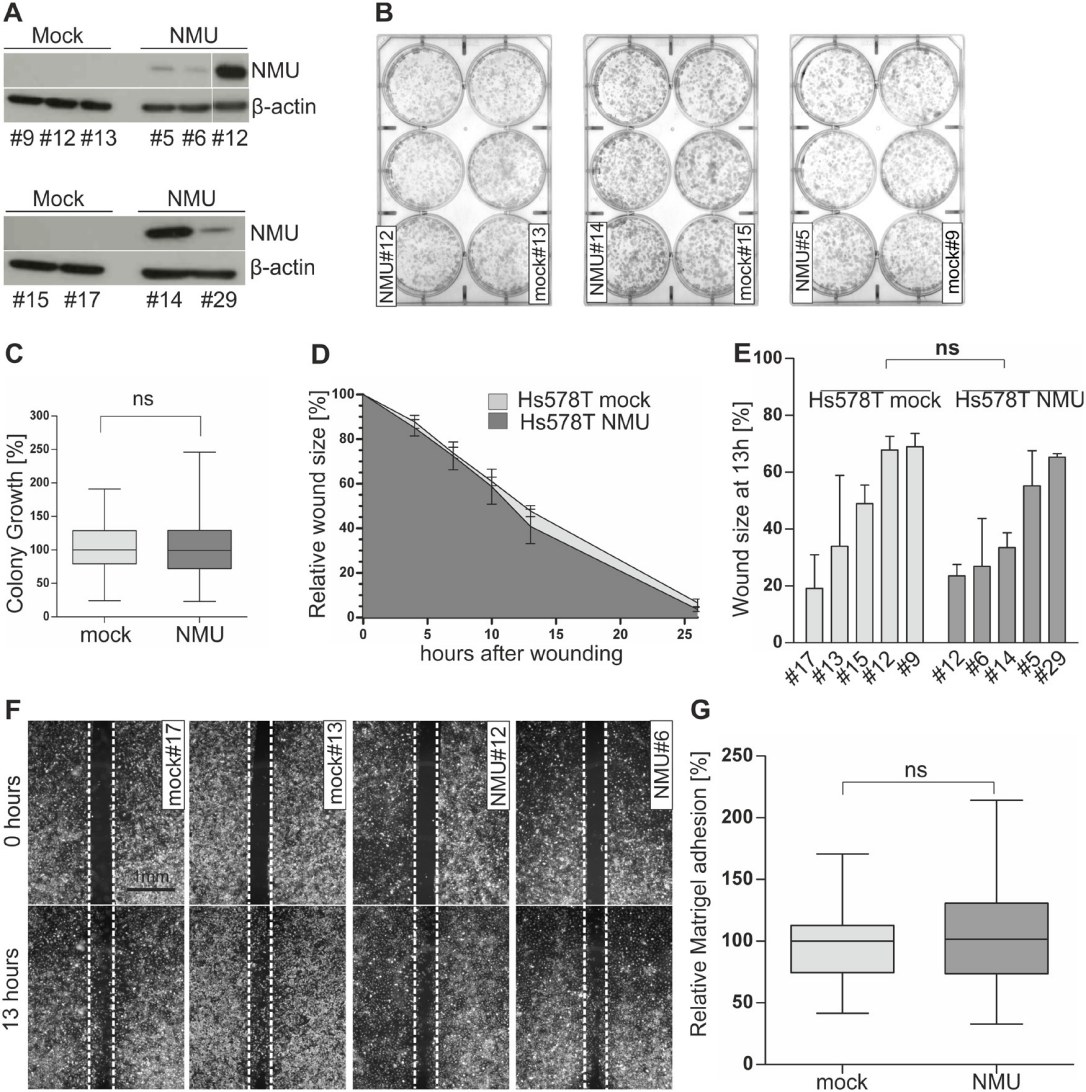
Ectopic NMU expression has no effect on NMUR2-negative Hs578T cells **(A)** NMU protein expression of stably transfected independent Hs578T mock (#9, #12, #13, #15, #17) and NMU (#5, #6, #12, #14, #29) clones. **(B)** Representative 6-well plates containing stable Hs578T mock and NMU clones are shown 2 weeks after cell seeding. **(C)** Densitometrical evaluation of 2D colony growth after 2 weeks. Box plot presents median colony growth of triplicate experiments for stably transfected independent Hs578T mock (n=5) and NMU (n=5) clones. Horizontal lines: grouped medians. Boxes: 25-75% quartiles. Vertical lines: range, minimum and maximum, ns: not significant (Mann-Whitney-U test). **(D)** Comparison of cell migration of stably transfected Hs578T mock (n=5) and NMU (n=5) clones analyzed by monolayer wound healing assay over 26 hours. Vertical lines: standard deviation of triplicates. Cell-free area at hour 0 was set 100% and used for standardization. **(E)** Detailed comparison of wound closure for each clone after 13h, vertical lines: standard deviation of triplicates, ns: not significant (Mann-Whitney-U test). **(F)** Wound documentation by phase contrast microscopy at time point 0 and 13h after start of experiment. Scale bar: 1000μm. **(G)** Cell-matrix adhesion of stably transfected Hs578T mock (n=5) and NMU (n=5) clones was measured colorimetrically. Box plot shows median values of 3 independent experiments. Horizontal lines: grouped medians. Boxes: 25-75% quartiles. Vertical lines: range, minimum and maximum, ns: not significant (Mann-Whitney-U test).

In light of the retrospective data indicating a notable impact of NMU in dependency of NMUR2 expression, we next aimed at analyzing the functional impact of NMU in breast cancer by generating two different *in vitro* tumor models reflecting an NMUR2-positive and NMUR2-negative background, respectively. Interestingly, as already observed in breast tumors, we clearly confirmed higher *NMU* mRNA expression in basal-like breast cancer cell lines compared to luminal-like cell lines (Figure 4B). In order to generate stable gain-of-function *in vitro* models over-expressing a full-length *NMU* cDNA, the basal-like Hs578T and the luminal-like, HER2-positive SKBR3 cell line were chosen as adequate *in vitro* tumor models showing marginal levels of *NMU* mRNA (Figure 4A) and no detectable NMU protein (Figure 4C). While SKBR3 cells expressed *NMUR2* as well as *NTSR1* mRNA and lacked the expression of *NMUR1* and *GHSR*, basal-type Hs578T cells expressed none of the potential NMU receptors (Figure 4D-4E). Subsequent to the stable transfection, ectopic NMU over-expression in single-cell clones was confirmed by real-time PCR (Supplementary File 2) and western blotting (Figure 5A and 6A).

Based on these two *in vitro* models, we started functional analyses to decipher the biological role of NMU in dependency of NMUR2 expression in breast cancer cells. Using a 2D colony formation assay over two weeks, we clearly demonstrated an impaired colony formation capability of SKBR3 NMU clones compared to mock vector transfected controls (Figure 5B). Densitometric evaluation revealed a highly significant (P < 0.001) median growth reduction of SKBR3 NMU single-cell clones (Figure 5C). Setting the median colony growth of controls to 100%, we observed a relative growth reduction of NMU clones by 46% to 54%. No significant effect of ectopic NMU expression on colony growth was noted for Hs578T clones (Figure 6B and 6C). Similar effects were observed by using the XTT cell viability assay (Supplementary File 3). To examine if the observed impact of NMU expression in SKBR3 cells on cell growth was due to enhanced apoptosis, we measured the activity of the effector caspases 3 and 7. Since no significant difference in the caspase 3/7 activity between SKBR3 NMU and mock clones was documented, we assumed an influence of NMU on cell proliferation in SKBR3 cells (Figure 5D).

To compare the effects of NMU expression on cell motility between NMUR2-positive and NMUR2-negative breast tumor cells, a monolayer wound healing assay was performed. Concerning the NMUR2-positive SKBR3 NMU gain-of-function model, we noted an accelerated wound closure of NMU clones compared with controls (Figure 5E and 5F). The average difference in wound size between NMU and mock clones was significant at each single time point over 6 days measurement and reached its peak at day 4 after wounding (Δ_day4_=19.5%, P < 0.001). Micrographs of magnified wound areas 4 days after scratching are shown in Figure 5G. No impact of forced NMU expression on cell migration was noted for the NMUR2-negative Hs578T NMU gain-of-function model (Figure 6D-6F).

As changes in tumor cell migration are often accompanied by alterations in tumor cell adhesion, we analyzed for the first time whether ectopic NMU expression alters cell-matrix adhesion of tumor cells. In the NMUR2-positive SKBR3 tumor model, we indeed observed a slight but reproducible decrease in median cell-Matrigel adhesion of NMU clones by 11.3 % compared to mock controls (Figure 5H, P < 0.05). Again, no difference in cell-Matrigel attachment was observed between NMUR2-negative Hs578T NMU and corresponding mock clones (Figure 6G).

Taken together, we showed for the first time that NMU reduced cell growth and cell-Matrigel adhesion of breast cancer cells and validated its promoting influence on breast cancer cell migration. These data further suggest for the first time that the observed effects of NMU on breast cancer cells are possibly mediated by the receptor NMUR2.

### Identification of a NMU-associated gene signature in NMUR2-positive SKBR3 breast cancer cells

**Figure 7:**
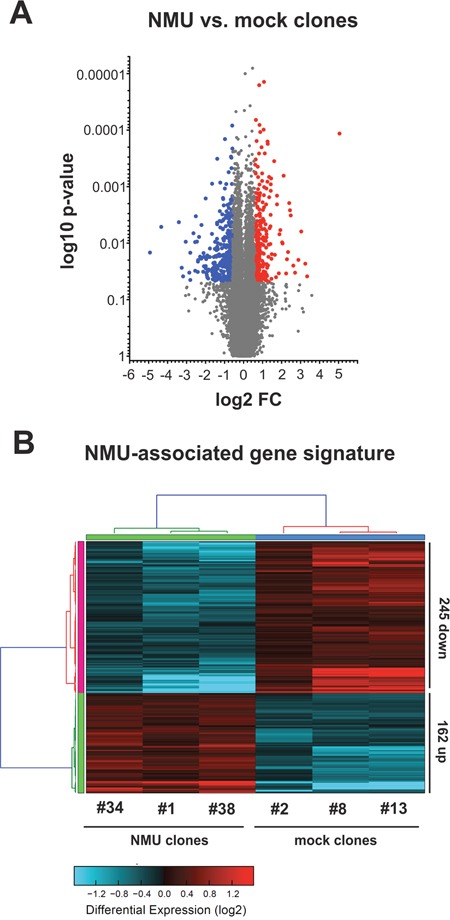
NMU-associated gene signature in NMUR2-positive breast cancer cells Comparison of gene expression profiles of SKBR3 clones expressing NMU and mock-transfected SKBR3 breast cancer cells. **(A)** Volcano plot analysis illustrating the whole range of gene expression of NMU and mock clones. Significantly up- (red dots) and down-regulated genes (blue dots) are highlighted. **(B)** Heatmap showing the NMU-associated 407-gene signature: significantly (*P* < 0.05) differentially expressed (*red*: up-regulated; *blue*: down-regulated) genes with a minimal change in expression by 1.5-fold.

To elucidate signaling pathways and genes that are regulated by forced NMU expression, we performed a comprehensive whole genome expression analysis for NMU in breast cancer employing the luminal-like, NMUR2-positive SKBR3 model. Using the Affymetrix GeneChip Human Gene 2.0 ST Array, we performed a whole transcript expression analysis of >45,000 RefSeq transcripts of three independent stably transfected SKBR3 NMU over-expressing and three independent mock single-cell clones. By applying a class comparison analysis between control cell populations (mock clones) and NMU-transfected clones (NMU clones) we aimed at identifying the strongest co- and anti-regulated genes which met the following criteria: Significantly (P < 0.05) differentially expressed with a minimal change in expression by 1.5-fold. Significantly up- and down-regulated genes are highlighted in the volcano plot (Figure 7A) and are further listed in the supplements (Supplementary File 4). The NMU-associated 407-gene signature in NMUR2-positive SKBR3 breast cancer cells is shown as heatmap in Figure 7B.

### Crosstalk of NMU with WNT superfamily signaling in NMUR2-positive SKBR3 breast cancer cells

In order to understand the function and the biological processes affected by NMU, we conducted a gene ontology (GO) analysis (Tables 2 and 3). In line with recently published studies, we found significant associations between NMU expression and previously described biological processes (BP) involving NMU, e.g. bone formation, regulation of blood pressure, inflammation and control of energy homeostasis/metabolic processes [3, 23]. Additionally, over-represented annotations indicate that cancer-relevant pathways are modulated by forced NMU expression, thereby affected by NMU signaling crosstalk. We identified significant associations with Ephrin receptor (P < 0.01), WNT (GO:0090090 and GO:0030177; P < 0.001 and P < 0.05), TGFβ (GO:0060395 and GO:0030511, for both P < 0.01), ERK (P < 0.01), and Smoothened signaling (P < 0.05) (Tables 2 and 3).

**Table 2:** Selected GO categories up-regulated in SKBR3 NMU over-expressing clones

GO category	GO name	GO type	Genes changed	Z-Score	Fisher exact P
GO:0045671	Negative regulation of osteoclast differentiation	BP	14.3%	5.6801	0.0017
GO:0003071	Renal system process involved in regulation of systemic arterial blood pressure	BP	14.3%	5.6801	0.0017
GO:0090090	Negative regulation of canonical WNT receptor signaling pathway	BP	7.2%	5.2387	0.0004
GO:0031532	Actin cytoskeleton reorganization	BP	9.8%	5.2046	0.0012
GO:0048013	Ephrin receptor signaling pathway	BP	9.7%	4.4832	0.0052
GO:0040012	Regulation of locomotion	BP	3.2%	4.4050	0.0002
GO:0001816	Cytokine production	BP	6.3%	3.8590	0.0061
GO:0051492	Regulation of stress fiber assembly	BP	7.3%	3.7315	0.0113
GO:0042127	Regulation of cell proliferation	BP	2.1%	3.4533	0.0018
GO:0045913	Positive regulation of carbohydrate metabolic process	BP	6.3%	3.3407	0.0173

Abbreviations: GO: gene ontology; BP: biological process

**Table 3:** Selected GO categories down-regulated in SKBR3 NMU over-expressing clones

GO category	GO name	GO type	Genes changed	Z-Score	Fisher exact P
GO:0006629	Lipid metabolic process	BP	4.3%	7.7679	< 0.0001
GO:0031018	Endocrine pancreas development	BP	11.9%	5.3896	0.0005
GO:0060395	SMAD protein signal transduction	BP	17.6%	5.3312	0.0022
GO:0007015	Actin filament organization	BP	6.7%	4.8038	0.0003
GO:0030511	Positive regulation of transforming growth factor beta receptor signaling pathway	BP	13.6%	4.5523	0.0047
GO:0070372	Regulation of ERK1 and ERK2 cascade	BP	6.3%	4.2688	0.0010
GO:0042127	Regulation of cell proliferation	BP	2.8%	3.6925	0.0008
GO:0022407	Regulation of cell-cell adhesion	BP	6.2%	3.3360	0.0090
GO:0030177	Positive regulation of WNT receptor signaling pathway	BP	5.3%	2.5922	0.0318
GO:0008589	Regulation of smoothened signaling pathway	BP	6.1%	2.5643	0.0417

Abbreviations: GO: gene ontology; BP: biological process

In consideration of a highly significant NMU-mediated modulation of members of the WNT signaling in SKBR3 breast cancer cells (Tables 2 and 3) and a previous association of the WNT receptor cascade with NMU [25], we focused on this pathway in detail. A cartoon highlighting the pattern of expression changes in Frizzled ligands, the WNT antagonist DKK1, WNT receptors, canonical WNT targets and non-canonical WNT effectors is illustrated in Supplementary File 5.

**Figure 8:**
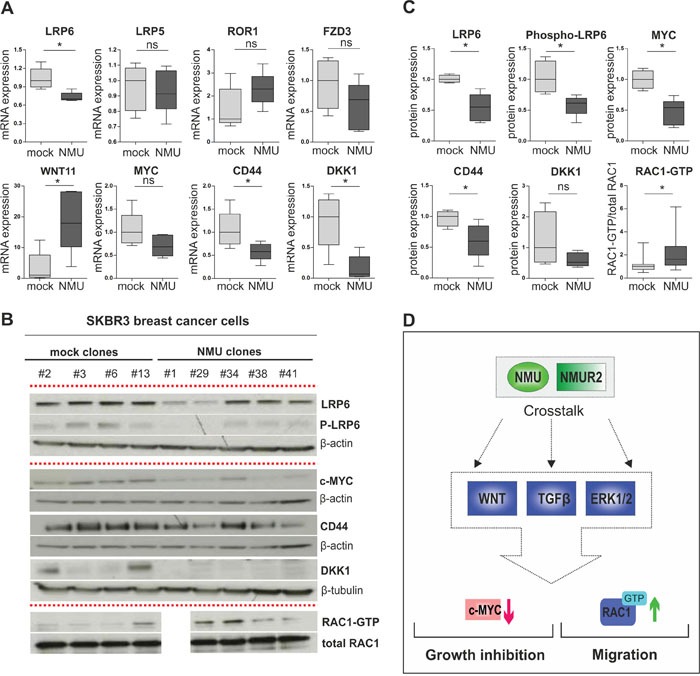
NMU signaling modulates the WNT receptor pathway in NMUR2-positive SKBR3 breast cancer cells **(A)** Real-time PCR-based validation of candidate NMU downstream genes in independent stably transfected SKBR3 NMU (n=5) and mock clones (n=5). * *P* < 0.05; ns: not significant (Mann-Whitney-U test). **(B)** Representative western blots showing differential protein expression of candidate NMU downstream genes in independent stably transfected SKBR3 NMU and mock clones. Loading controls: β-actin, β-tubulin, total RAC1. All experiments were performed in triplicate. **(C)** Densitometrical evaluation of the western blot results shown in B depicted as box plots. Box plot showing RAC1-GTP in relation to total RAC1 amounts in SKBR3 NMU (n=4) and mock clones (n=4), combines data of three independent experiments. * *P* < 0.05; ns: not significant (Mann-Whitney-U test). **(D)** Hypothetical model of NMU's oncogenic role in dependency of NMUR2 in breast cancer: crosstalk of NMU signaling with WNT, TGFβ and ERK cascade results in decreased expression of the canonical WNT target MYC and enhanced activation of the non-canonical WNT/planar cell polarity (PCP) pathway effector RAC1 among others, contributing to growth inhibition and promotion of cell migration.

In a subsequent real-time PCR-based validation step using a larger cohort of independent stably transfected SKBR3 NMU (n=5) and mock single-cell clones (n=5), expression of WNT signaling members downstream of NMU was clearly demonstrated (Figure 8A). In line with the microarray data, we observed down-regulation of the canonical WNT co-receptor LRP6 (median FC: 1.4), the canonical WNT targets CD44 (median FC: 1.7) and MYC (median FC: 1.5) as well as of the WNT antagonist DKK1 (median FC: 14.7) in SKBR3 NMU clones compared to empty vector controls. Moreover, we verified NMU-mediated up-regulation of the non-canonical WNT co-receptor ROR1 (median FC: 2.3) as well as the Frizzled ligand WNT11 (median FC: 17.9).

Finally, we aimed to identify protein changes of core WNT components in SKBR3 NMU clones possibly contributing to manifestation of the motile, slow-growing phenotype observed. As such expression loss of total LRP6 protein and the activated phosphorylated form (Ser1490) of the canonical WNT co-receptor (for both P < 0.05, Figure 8B and 8C) was found. Decreased protein level of the known TCF/LEF-regulated WNT targets MYC and CD44 was present as well. Importantly, significantly increased activation of the WNT/PCP effector RAC1 in SKBR3 NMU clones was observed compared to control cells lacking NMU expression (for all P < 0.05, Figure 8B and 8C).

In sum, our results propose the following hypothetical model of NMU's oncogenic role in dependency of NMUR2 expression in breast cancer: crosstalk of NMU signaling with several cancer-relevant pathways, e.g. WNT, TGFβ and ERK cascade results in decreased expression of the canonical WNT target MYC and enhanced activation of the non-canonical WNT/planar cell polarity (PCP) pathway effector RAC1 among others, possibly contributing to growth inhibition and promotion of cell migration (Figure 8D).

## DISCUSSION

NMU has previously been associated with cancer, especially disease progression (metastasis) of several tumor entities, by enhancing motility and invasiveness of cancer cells [16–19, 21]. Interestingly, also other members of the neuromedin family of neuropeptides, such as neuromedin B (NMB) and gastrin releasing peptide (GRP), have been identified as cancer-promoting factors in human tumors [26, 27]. Until recently a possible involvement of NMU in breast carcinogenesis has not been analyzed. Rani and colleagues described the neuropeptide as candidate drug response biomarker for HER2-targeted therapies and interestingly, as putative therapeutic target to reduce metastatic spread of breast cancer cells [20]. Nevertheless, a comprehensive expression analysis of NMU and its potential receptors in the different breast cancer subtypes as well as a systematic evaluation of NMU-modulated cancer-relevant signaling pathways and associated molecules is still missing.

We conducted, for the first time, a profound subtype-specific expression analysis for NMU in human breast cancer tissue samples and healthy breast tissue specimens. We demonstrated that *NMU* mRNA expression is significantly up-regulated in all molecular breast cancer subtypes (except for normal-like cases) compared to normal breast tissue controls. Highest levels of *NMU* expression were detected in HER2-enriched and basal-like breast carcinomas as well as advanced tumors of larger size, proposing a possible positive selection of NMU expressing cancer cells in advanced tumor stages. Interestingly, a significant correlation between *NMU* expression and adverse overall survival was noticed in advanced nodal-positive (pN1-2) carcinomas supporting the hypothesis of a potential critical role for NMU in breast cancer progression. Moreover, we also provide first-time evidence that NMU protein is expressed in human breast cancer cells using immunohistochemistry and western blot analysis. We were able to detect NMU protein only in those cases showing very high levels of *NMU* mRNA expression either implicating minor sensitivity of the antibody used and/or a low half-life of the protein [3]. However, in line with our mRNA data, these preliminary qualitative findings on protein level indicate an up-regulation of NMU protein in breast cancer cells compared to expression in associated normal breast epithelium.

NMU expression has been demonstrated as potential prognostic factor of an unfavorable prognosis in non-small cell lung cancer and endometrial carcinomas [17, 21]. Concerning breast cancer, the overall prognostic relevance of NMU ligand has recently been reported by Rani and colleagues [20]. However, this study did not evaluate the association between NMU expression and patient survival in a receptor dependent manner that may help to understand the varying impact of NMU on the different breast cancer subtypes. Here, univariate analyses demonstrated that high *NMU* mRNA expression predicted poor prognosis only in carcinomas showing high *NMUR2* expression whereas no significant association of NMU expression with shorter overall survival in any other receptor combination was observed. Interestingly, our study revealed that *NMUR2* was expressed in all breast cancer subtypes except for basal-type carcinomas almost completely lacking *NMUR2* transcript. These data are in concordance with the observation made by Rani and co-workers showing a prognostic impact of NMU expression in all breast cancer subtypes but basal-like carcinomas.

In light of the retrospective data suggesting a potential oncogenic role for NMU in a NMUR2-positive background, we next aimed at analyzing the functional impact of NMU on breast cancer cells in dependency of NMU receptor expression. In accordance with the clinical impact of NMU, we observed NMU-associated phenotypical effects in the luminal-like *NMUR2*-expressing SKBR3 model, but not in basal-like *NMUR2*-negative Hs578T cells. NMU expression in SKBR3 cells caused increased cellular motility, an altered cell-matrix adhesion and interestingly, a reduced proliferative activity compared to empty vector control single-cell clones. In accordance with our findings, Rani and colleagues described NMU-mediated promotion of cell motility and invasiveness of SKBR3 cells *in vitro* as well. Moreover, they interestingly showed an increased anoikis resistance of these cells, but did not report on a change in adhesive properties or cell growth [20]. It has already been described for various cancer progression-associated factors such as AGR2, SIP1 and YB1 [28–30] to promote on the one hand tumor cell migration while inhibiting tumor growth on the other hand as demonstrated for NMU in our SKBR3 *in vitro* model. It is appreciated that disseminating tumor cells activate a cellular program resulting in an enhanced motile/invasive capacity and reduced proliferation (tumor cell dormancy) [31, 32]. These slow-growing, mobile cells have the ability to evade anoikis [33] as well as to survive therapeutic interventions primarily targeting actively dividing cells [31]. Similar effects of NMU on cell migration, invasion and anoikis resistance were seen in basal-like NMUR2-expressing HCC1954 cells. However, in a mouse xenograft experiment using these cells a growth-supporting effect of NMU was reported [20]. These data are in agreement with the observations made by Wu and colleagues reporting a growth-inhibitory effect of NMU on T24 bladder cancer cells in an anchorage-dependent *in vitro* assay while NMU stimulated growth of the same cells in a mouse xenograft experiment *in vivo* suggesting a dependence of NMU-mediated promotion of tumor formation on the tumor microenvironment [16]. In line with two recent studies, also NMUR2 was identified as the receptor responsible for NMU-mediated enhancement of cellular motility and invasiveness in human pancreatic and endometrial cancer cells [18, 21]. Taken together, the data presented here suggest for the first time that NMU-associated oncogenic effects on breast cancer cells are possibly mediated by the receptor NMUR2. Further, in combination with the observations made by Rani et al. [20] our data suggest that NMU might contribute to the development of cancer cells with an increased ability to disseminate from the primary tumor and to metastasise.

As a systematic analysis approach of signaling pathways and associated molecules affected by NMU in breast cancer is still missing we applied the luminal-like, NMUR2-positive SKBR3 gain-of-function model to perform a comprehensive whole genome expression analysis. We identified a NMU-associated gene signature as more than 400 genes were regulated by NMU expression. In accordance with previous studies [3, 23], we found significant associations between NMU expression and physiological processes, e.g. bone formation, regulation of blood pressure and inflammation. Moreover, NMU significantly affected modulation of several cancer-relevant pathways, i.e. WNT, Ephrin receptor, TGFβ, ERK and Smoothened signaling of which the WNT [25] and ERK [34] cascade have been linked to NMU signaling before.

In the following we focussed on key components of the WNT signaling cascade keeping in mind that an isolated analysis of this cascade might be difficult regarding its crosstalk with further pathways modulated by NMU signaling, e.g. TGFβ [35, 36] and ERK1/2 [37]. As such we noted down-regulation of the canonical WNT/β-catenin target MYC [38] among other TCF/LEF-regulated genes in NMU over-expressing SKBR3 clones. Reduced expression of the important transcription factor MYC that is known to regulate cellular processes such as proliferation and apoptosis [39] fits the observed growth reduction of SKBR3 NMU clones. In further support of our findings, two previous studies propose a possible stimulation of cell proliferation and a parallel inhibition of cell motility and invasiveness by MYC in breast cancer cells [40, 41]. In turn, we demonstrated a significantly increased activation of the small G-protein RAC1, an effector of the WNT/PCP cascade [42] proven to enhance motility and invasiveness of cancer cells [43, 44]. Interestingly, Lin and co-workers recently described a NMU-NMUR2-mediated modulation of CD44 and RAC1 expression in endometrial cancer cells [21].

In summary, we showed for the first time that NMU expression is up-regulated in breast carcinomas of all molecular subtypes and advanced tumor stages compared to normal breast tissue expression. Further, our data propose that the receptor NMUR2 is likely to mediate NMU-related effects on breast cancer cells. We provide evidence that NMU promotes motile characteristics while suppressing growth of NMUR2-positive breast cancer cells potentially contributing to progression of a subset of breast cancer cases. Our data suggest a crosstalk of NMU signaling with several cancer-relevant pathways including the WNT receptor cascade resulting in an increased activation of the WNT/PCP effector RAC1 as an indicator for enhanced cancer cell motility and down-regulation of the canonical WNT target MYC among others. Given the fact that NMU exerts its actions via cell surface receptors makes it a potential druggable target. Future molecular studies focusing in detail on the responsible NMU receptor(s) and downstream factors of the NMU-driven signaling network are needed and may provide novel personalized strategies to prevent metastatic spread of a subset of breast cancer cases.

## MATERIALS AND METHODS

### Cell lines

The human breast cancer cell lines SKBR3 and Hs578T were originally obtained from the American Type Culture Collection (Rockville, MD, US) and cultured under recommended conditions. Cell lines were regularly tested for mycoplasma infection using the PCR-based *Venor^®^ GeM Mycoplasma Detection Kit* (Minerva Biolabs, Berlin, Germany).

### Generating stable single-cell clones

All transfections were performed using *FuGene HD Transfection Reagent* (Roche, Mannheim, Germany) following the manufacturer's instructions and either the pT-REx-DEST30 vector construct containing the full-length human *NMU* cDNA (Source BioScience LifeSciences, Nottingham, England) or the pT-REx-DEST30 empty vector (Invitrogen, Carlsbad, CA, USA) as control. Selection of stable NMU and empty vector single-cell clones was achieved by culturing SKBR3 and Hs578T cells in complete culture medium containing 0.8 mg/mL and 0.6 mg/mL G418 (Life Technologies, Darmstadt, Germany) respectively for at least two weeks to ensure genomic cDNA integration. Afterwards, isolated clones were analyzed by both real-time PCR and western blotting for expression of NMU.

### Clinical specimens

Cryoconserved and formalin-fixed, paraffin-embedded (FFPE) tumorous and normal breast tissue samples analyzed in this study were obtained from the tumor bank of Euregional comprehensive Cancer Center Aachen (ECCA), now part of the RWTH centralized biomaterial bank (RWTH cBMB). All patients gave written informed consent for retention and analysis of their tissue for research purposes according to local Institutional Review Board (IRB)-approved protocols (approval no. EK-206/09) of the Medical Faculty at RWTH Aachen University. After surgery, tumor material was immediately snap-frozen in liquid nitrogen. Sections stained with haematoxylin and eosin, were prepared for assessing the percentage of tumor and normal epithelial cells, respectively. Only cryoconserved tumor samples containing more than 70% tumor cells, and normal samples containing at least 30% epithelial cells as determined by a pathologist (W.A.), were selected for RNA analysis. Patient characteristics for fresh frozen tumor samples are shown in the supplements (Supplementary File 1).

### TCGA patients‘ data set

Raw Illumina HiSeq expression data for *NMU, NMUR1, NMUR2*, *NTSR1* and *GHSR* as well as the corresponding clinical data of the breast cancer samples (n=1082) and normal tissues (n=113) analyzed, were used from The Cancer Genome Atlas (TCGA) [24]. Using sample IDs (see Supplementary File 6), the expression data for *NMU, NMUR1*, *NMUR2, NTSR1* and *GHSR* of breast cancer specimens can be downloaded at the cBio Cancer Genomics Portal [45] (http://www.cbioportal.org), whereas the corresponding clinical data are available at The Cancer Genome Atlas Data Portal (https://gdc.cancer.gov/).

### RNA extraction and reverse transcription PCR

Total RNA from cryoconserved tissues was isolated using the standard procedure for *TRIzol®* (Invitrogen, Carlsbad, CA, USA) RNA extraction. Extracted RNA was quantified using the *NanoDrop ND1000* spectrophotometer (Thermo Scientific, Waltham, MA, USA). The A260 nm/A280 nm ratio was generally between 1.9 and 2.0. Subsequently, cDNA was synthesised using 1μg of total RNA and the reverse transcription system (Promega, Madison, WI, USA) according to the manufacturer's instructions. Briefly, after heat denaturing of RNA (in 8.9μl RNase-free water) for 10min at 70°C, 11.1μl of a mix containing 15U of *AMV* reverse transcriptase, 20U RNase inhibitor and each 0.5μg of both oligo(dT)_15_ and random primers was added to the RNA and the reaction tube was subsequently incubated for 10min at RT, followed by the synthesis step for 15min at 42°C. After cDNA synthesis, enzyme was heat inactivated by incubation for 5min at 95°C. cDNA was stored at -20°C until use.

### Semi-quantitative real-time PCR

cDNAs were amplified by semi-quantitative real-time PCR using *SYBR Green PCR mix* (Bio-Rad Laboratories, München, Germany) and the iCycler *IQ5* (Bio-Rad Laboratories) as described previously [46]. Gene-specific primer sets were designed by using *Primer3web* software (version 4.0.0) (http://primer3.ut.ee/). All reactions were performed in triplicates including negative controls without cDNA. Specificity of amplification products was confirmed by size estimation on agarose gels and melt curve analysis. Obtained data were analyzed using the comparative Ct (threshold cycle) method. Complete reaction conditions, primer sequences and lengths of amplicons are listed in Supplementary File 7.

### Western blotting

Total cell protein lysates of human breast cancer cell lines and tissue specimens were obtained by sonification of cells/tissues in an appropriate volume of 1×*NuPAGE LDS Sample Buffer* (Invitrogen, Carlsbad, CA) supplemented with 50mM dithiothreitol (Life Technologies, Darmstadt, Germany). Heat denatured samples were loaded on 4-12% gradient gels (NuPAGE; Invitrogen) and then transferred onto 0.2μm PVDF membranes (Whatman, Dassel, Germany) (1h, 100V) for immunodetection. Blots were blocked in TRIS-buffered saline (TBS) containing 0.1% Tween-20 (TBS-T) and either 5% non-fat dry milk (Merck, Darmstadt, Germany) or BSA (Roth, Karlsruhe, Germany) for 1h at room temperature. Blocked blots were then incubated with the primary antibody overnight at 4°C, diluted in blocking solution either containing 5% non-fat dry milk or BSA. The following primary antibodies were used: NMU (1:250, HPA025926, Atlas Antibodies, Stockholm, Sweden), β-actin (1:5000, A5441, Sigma-Aldrich, Deisenhofen, Germany), β-tubulin (1:500, ab6046, Abcam, Cambridge, UK), CD44 (1:1000, 156-3C11), MYC (1:1000, D84C12), DKK1 (1:1000, 4687), LRP6 (1:1000, C47E12), P-LRP6 (1:1000, 2568) (all Cell Signaling, Danvers, MA, USA). After washing three times (TBS + 0.05% Tween-20), blots were incubated with secondary peroxidase-conjugated antibodies (DAKO, Glostrup, Denmark) diluted in blocking solution containing 5% non-fat dry milk for 1h at room temperature. After washing three times (TBS + 0.05% Tween-20), antibody detection was accomplished with Pierce ECL Western blotting Substrate (Thermo Scientific, Rockford, IL, USA).

### RAC1 pulldown

Measurement of RAC1 activation in stable SKBR3 NMU and mock clones was achieved by using the *Active RAC1 Detection Kit* (#8815, Cell Signaling, Danvers, MA, USA) according to the manufacturer's instructions. In brief, single-cell clones were cultured in G418 containing growth medium for 48h until a cell confluency of 80% was reached. After cell lysis, 550μg of total cell protein lysate of each clone were mixed with 20μg of GST-PAK1-PBD, selectively capturing (active) RAC1-GTP. Glutathione matrix-immobilized RAC1-GTP was finally eluted in SDS sample buffer supplemented with DTT, heat denatured (5min, 95°C) and analyzed by western blot employing a RAC1-specific antibody (1:1000). For normalization of RAC1-GTP corresponding signals, the amount of total cellular RAC1 protein in each lysate was detected and the ratio of both signals was calculated.

### Immunohistochemistry

FFPE sections (3μm) were dried overnight (37°C). Afterwards, paraffin was removed in xylene and tissue sections were rehydrated using a descending alcohol series. Heat-induced epitope retrieval was performed in 10mM citrate buffer (pH 6.0) for 30 minutes using a water bath (98°C). Immunohistochemical analysis was carried out using an established bench protocol for the Dako REAL™ Detection System, Peroxidase/DAB+, Rabbit/Mouse kit (K5001; Dako, Glostrup, Denmark). In short, cooled down FFPE sections were incubated with Dako REAL™ Peroxidase-Blocking Solution (S2023, 5min) followed by a washing step and incubation with an NMU-specific antibody (1:20; HPA025926, Atlas Antibodies, Stockholm, Sweden) for 45 minutes at room temperature. After three washing steps, biotinylated secondary antibody and peroxidase-conjugated streptavidin solution were applied (in each case 15 minutes, room temperature) before addition of DAB chromogen solution of the Dako kit (5min). NMU protein staining was evaluated by an experienced pathologist (S.V.S.).

### Colony formation assay

Cell growth of stable SKBR3 and Hs578T NMU and mock clones was analyzed by conducting a 2D colony formation assay. Briefly, cells of independent clones were seeded in six-well plates (1,000 cells/well) containing growth medium supplemented with G418. Medium was replaced every two days. After two weeks cultivation (20% O_2_, 5% CO_2_, 37°C), cells were fixed and stained using a 0.5% crystal violet staining solution (80% methanol, 10% formaldehyde, 10% ddH_2_O). Densitometrical evaluation of photographs was accomplished by using ImageJ Software (1.45, National Institute of Health, USA). Experiments were performed in triplicate.

### XTT cell viability assay

For cell viability analysis the *XTT cell proliferation kit II* from Roche (Mannheim, Germany) was used. Briefly, cells of independent SKBR3 and Hs578T single-cell clones were seeded in 96-well plates (1,000 cells/well) containing growth medium supplemented with G418. Cell viability was determined at four different time points: 24, 48, 72 and 96 h after cell seeding. 50 μl of XTT working solution were added to each well and afterwards incubated for 4 h. Finally the absorbance was determined at 492 and 650 nm. Experiments were performed in triplicate.

### Apoptosis assay

Activity of the effector caspases 3 and 7 in stable SKBR3 NMU and mock clones, as indicator of apoptosis, was determined by using the *Apo-One® Homogeneous Caspase-3/7 Assay* (Promega, Mannheim, Germany) according to the manufacturer's instructions. Briefly, cells (1.5×10^4^) were seeded in 96-cell culture wells and incubated overnight (20% O_2_, 5% CO_2_, 37°C). Afterwards, staurosporine (final concentration 1μM, Sigma-Aldrich, Deisenhofen, Germany) was added to induce apoptosis. After 24h, lysis/substrate buffer was added leading to cleavage of the contained profluorescent caspase substrate Z-DEVD-R110 to create fluorescent rhodamine 110. The fluorescence signal, proportional to caspase 3/7 activity, was quantified by using an ELISA plate reader (excitation: λ=485 nm; emission: λ=577 nm). Experiments were performed in triplicate.

### Migration assay

Motility of stable SKBR3/Hs578T NMU and mock clones was assessed by performing a monolayer scratch wound healing assay. SKBR3 and Hs578T cells (8×10^4^ and 3.6×10^4^ respectively) were plated into six-wells containing single culture inserts (2×0.22cm^2^, 70μl, 500μm cell-free gap, Ibidi, Martinsried, Germany). After 24h the inserts were removed, generating a defined 500μm scratch. Cells were washed twice (phosphate-buffered saline) and cultured with growth medium. Images of cell-free areas were taken at the indicated time points with a CCD camera Colour View III (Olympus, Hamburg, Germany) fitted to a light microscope. Cell-free areas were quantified using ImageJ Software (1.45, National Institute of Health, USA) [47]. Experiments were performed in triplicate.

### Cell-matrix adhesion assay

Cell-matrix adhesion was assessed by coating six-well plates with 10μg/ml Matrigel (BD Bioscience, Heidelberg, Germany). SKBR3 and Hs578T cells (3×10^5^ cells/well) were plated, incubated for 1h (20% O_2_, 5% CO_2_, 37°C) and gently washed three times with phosphate-buffered saline. Attached cells were fixed with 70% ethanol (10min) and stained with 0.1% crystal violet solution (20min). Cells were washed thoroughly with water and dried overnight. The dye was dissolved in 0.02% Triton X-100 in 100% isopropanol and carried over into a 96-well plate to measure the optical density at 590 nm.

### Gene expression profiling

Gene expression analysis of stably transfected SKBR3 NMU and mock clones was carried out by the IZKF Genomics-Facility (Interdisciplinary Center for Clinical Research Aachen within the medical faculty of the RWTH Aachen University) using the *GeneChip® Human Gene 2.0 ST Array* (Affymetrix, Santa Clara, CA, USA) in independent triplicates. Total RNA was isolated using the *TRIzol* method and quantified (Nanodrop). RNA quality was assessed using *RNA 6000 Nano Assay* with the *2100 Bioanalyzer* (Agilent, Santa Clara, CA, USA). Samples, each 300 ng total RNA, for the *GeneChip® Human Gene 2.0 ST Arrays* were prepared and hybridized to the arrays according to the Ambion whole-transcript expression and the Affymetrix whole-transcript terminal labeling and control kit manuals as described before [48]. Processed samples were hybridized to the *GeneChip® Human Gene 2.0 ST Arrays* at 45°C for 16 h with 60 rpms, washed and stained on a *Fluidics Station 450* (program: FS450 0002) and scanned on a *GeneChip® Scanner 3000 7G* (both Affymetrix). Raw image data were analyzed with *Affymetrix® Expression Console™* Software (Affymetrix, USA), gene expression intensities were normalized and summarized with robust multiarray average algorithm [49]. In order to identify genes differentially expressed between stable SKBR3 NMU and mock clones a class comparison analysis using *Affymetrix Transcriptome Analysis Console (TAC) 2.0* Software was performed. Differences were considered significant if the two-sided *P* value was < 0.05. To perform pathway over-representation analysis, data were analyzed with the software package *AltAnalyze* (version 2.0.8) [50] using Gene Ontology (GO) terms from the program *GO-Elite* [51]. The *GO-Elite* over-representation filtering parameters were a Z-score threshold of 1.96, a Fisher's exact test *P* value threshold of 0.05, and a number of changed genes threshold of 2. Gene expression was considered as changed if transcript levels between test (NMU) and control (mock) group were differential with a 1.5-fold change and a raw *P* value < 0.05. The microarray data from this publication have been submitted to the GEO repository and are available under accession number GSE75932.

### Statistical analysis

Statistical packages *SPSS 19.0* (SPSS, Chicago, IL, USA) and *GraphPad Prism 5.0* (GraphPad Software, La Jolla, CA, USA) were applied for data analysis. Differences were considered significant if the two-sided *P* values were < 0.05. To compare two groups the non-parametric Mann-Whitney *U*-test and for comparison of more than two groups the Kruskal-Wallis test was used. The Fisher's exact test was performed in order to correlate clinico-pathological parameters with *NMU* mRNA expression. Overall survival (OS) was measured from surgery until death and was censored for patients alive at the last follow-up using the univariate log-rank tests.

## SUPPLEMENTARY MATERIALS FIGURES AND TABLES







## Abbreviations

AGR2: anterior gradient 2
AMV: avian myeloblastosis virus
BP: biological process
BSA: bovine serum albumin
cBMB: centralized Biomaterial Bank
cDNA: complementary deoxyribonucleic acid
Ct: cycle threshold
ddH_2_O: double-destilled water
DKK1: Dickkopf 1
ECCA: Euregional comprehensive Cancer Center Aachen
ECL: enhanced chemiluminescence
ER: estrogen receptor
FC: fold change
FFPE: formalin-fixed, paraffin-embedded
FISH: fluorescence *in situ* hybridization
GHSR: growth hormone secretagogue receptor
GO: gene ontology
GST: glutathione S-transferase
HER2: human epidermal growth factor receptor 2
IHC: immunohistochemistry
IRB: Institutional Review Board
IZKF: Interdisciplinary Center for Clinical Research Aachen
LDS: lithium dodecyl sulfate
LEF: lymphoid enhancer-binding factor
mRNA: messenger ribonucleic acid
NMU: neuromedin U
NMUR1: neuromedin U receptor 1
NMUR2: neuromedin U receptor 2
NTSR1: neurotensin receptor 1
PCP: planar cell polarity
PCR: polymerase chain reaction
PVDF: polyvinylidene difluoride
PR: progesterone receptor
PAK1: p21 protein-activated kinase 1
RAC1: ras-related C3 botulinum toxin substrate 1
RT: room temperature
SDS: sodium dodecyl sulfate
SIP1: survival of motor neuron protein interacting protein 1
TBS: TRIS-buffered saline
TCF: T-cell factor
TCGA: The Cancer Genome Atlas
TGFβ: transforming growth factor β
TNBC: triple negative breast cancer
WNT: wingless-type MMTV integration site family
