# Supplementary material for: Oncogenic features of neuromedin U in breast cancer are associated with NMUR2 expression involving crosstalk with members of the WNT signaling pathway
Source: Oncotarget. 2017 Mar 11;8(22):36246–65. doi: 10.18632/oncotarget.16121 (PMC5482652; doi:10.18632/oncotarget.16121)
Supplement: Supplementary file 1 [file oncotarget-08-36246-s001.pdf]

Oncogenic features of neuromedin U in breast cancer are associated with NMUR2 expression involving crosstalk with members of the WNT signaling pathway

SUPPLEMENTARY FILES

Supplementary File 1: Clinico-pathological parameters of 62 breast cancer specimens analyzed in this study

Parameter	Categorization	n[a] analyzable	%
Age at diagnosis:	median 63.5 years (range 33-84)		
	<63.5 years	31	50.0
	≥63.5 years	31	50.0
Tumor size[b]			
	pT1	35	56.5
	pT2	25	40.3
	pT3	2	3.2
	pT4	0	0
Lymph node status[b]			
	pN0	34	54.8
	pN1-3	27	43.6
	unknown	1	1.6
Histological tumor grade[c]			
	G1	2	3.2
	G2	21	33.9
	G3	38	61.3
	unknown	1	1.6
Histological type			
	invasive ductal	55	88.7
	invasive lobular	5	8.1
	other	2	3.2
Estrogen receptor status			
	negative (IRS[d] 0-2)	18	29.0
	positive (IRS[d] 3-12)	42	67.7
	unknown	2	3.2
Progesterone receptor status			
	negative (IRS[d] 0-2)	20	32.3
	positive (IRS[d] 3-12)	39	62.9
	unknown	3	4.8
HER2 status[e]			
	negative	53	85.5
	positive	8	12.9
	unknown	1	1.6

[a]Only female patients with primary, unilateral, invasive breast cancer were included. [b]According to TNM classification by Sobin and Wittekind [52]. [c]According to Bloom and Richardson, as modified by Elston and Ellis [53]. [d]Immunoreactive score (IRS) according to Remmele and Stegner [54]. [e]Overexpression of the *ERBB2* gene (*HER2/neu*) was diagnosed analogously to the threshold of the DAKO-Score system based on IHC assay. Uncertain cases were additionally validated by FISH assay. Percentages may not sum-up to 100% due to rounding.

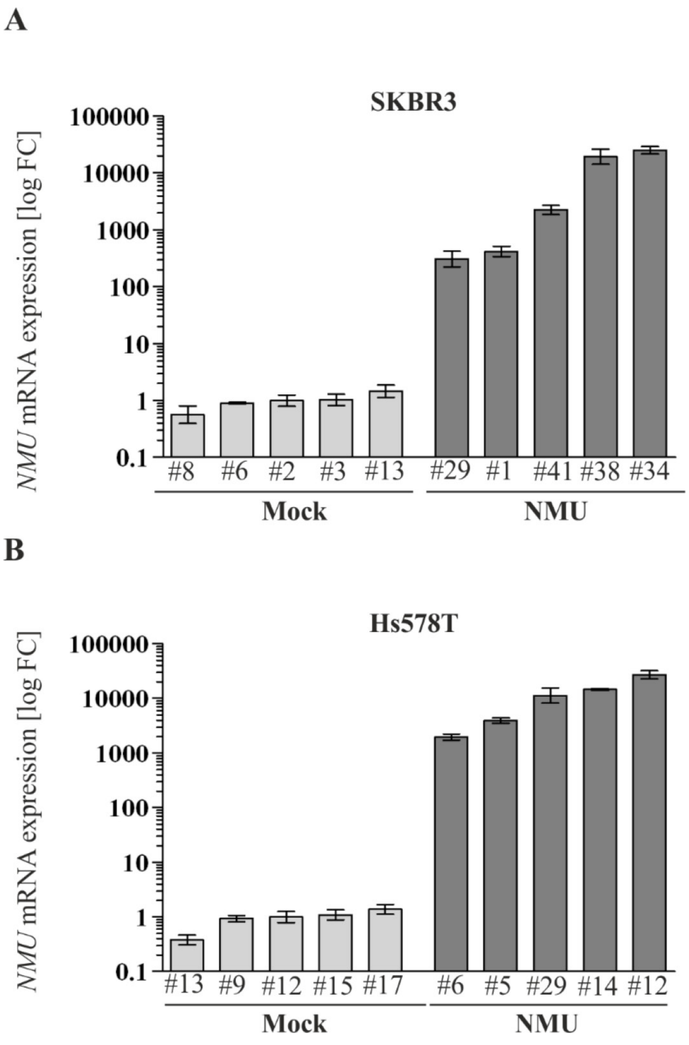

Supplementary File 2: *NMU* mRNA expression in stable single-cell clones. Real-time PCR-based *NMU* expression analysis in independent stably transfected NMU (n=5) and mock clones (n=5) of the **(A)** SKBR3 and **(B)** Hs578T gain-of-function *in vitro* model.

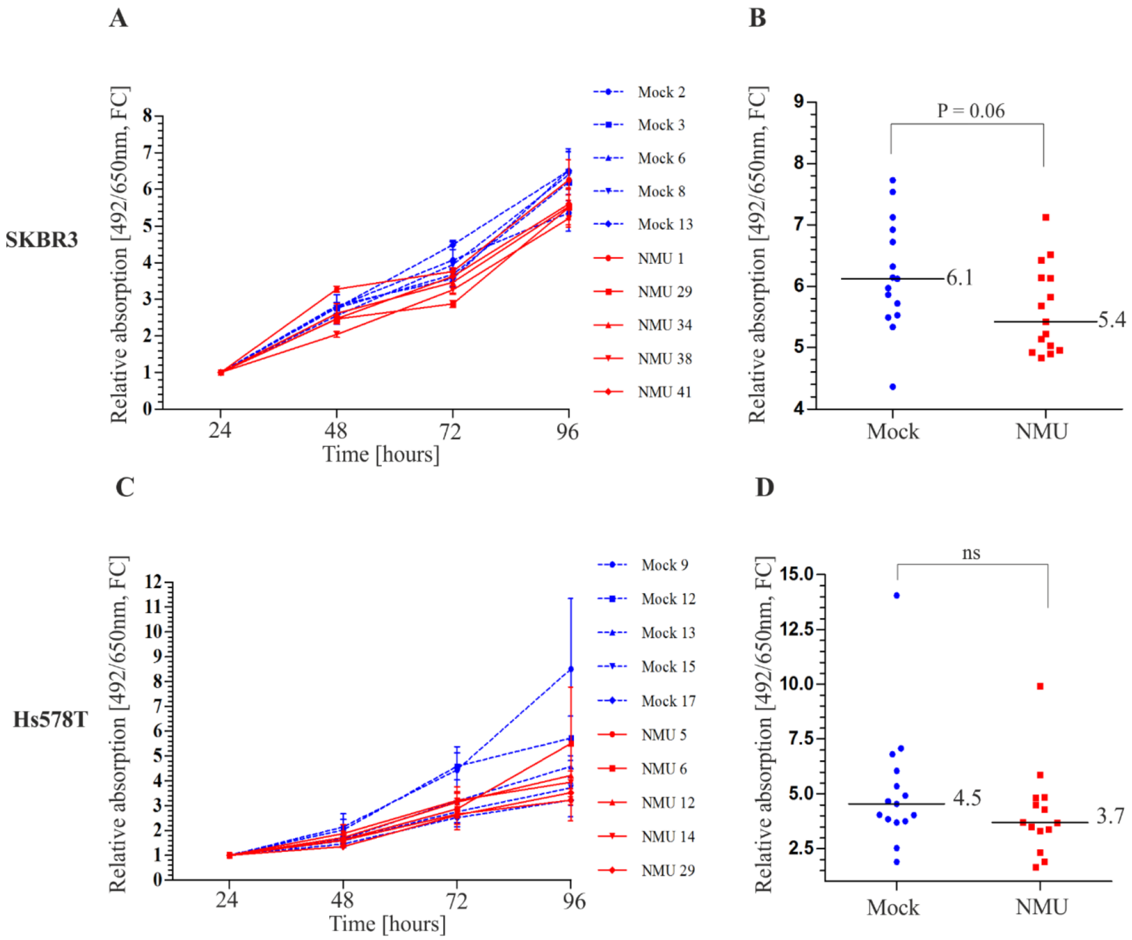

Supplementary File 3: XTT cell viability assay. Cell viability of independent NMU (n=5) and empty vector (n=5) clones of both *in vitro* models (SKBR3 and Hs578T) was determined colorimetrically (absorbance at 492/650nm) at four different time points: 24, 48, 72 and 96 h after cell seeding **(A** and **C)**. The baseline level at 24 h for each clone was set to 1. Experiments were performed in triplicate. Vertical lines: ± standard error of margin (SEM). Scatter plots showing the triplicate cell viability measurements for each clone at 96h **(B** and **D)**. Horizontal lines: grouped medians. Ns: not significant (Mann-Whitney-U test).

Supplementary File 4:

See Supplementary File 4

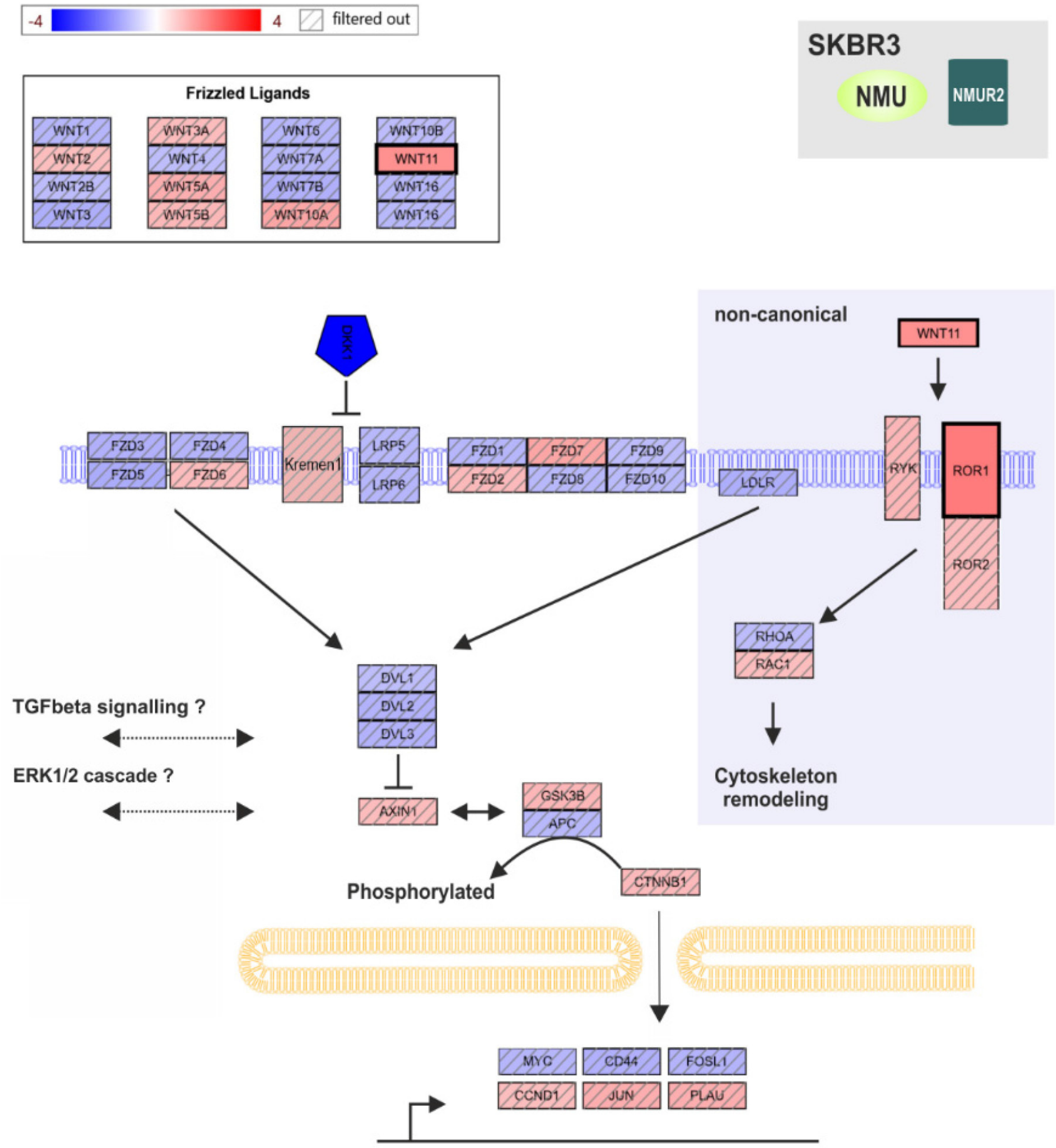

Supplementary File 5: NMU signaling modulates the WNT receptor signaling pathway in NMUR2-positive SKBR3 breast cancer cells. Simplified illustration of canonical and non-canonical WNT signaling components analyzed in SKBR3 NMU (n=3) and SKBR3 mock clones (n=3) by microarray analysis. Blue color: gene expression down-regulated, white color: expression not affected, red color: gene expression up-regulated in SKBR3 NMU clones compared to SKBR3 mock clones. Gene expression differences were considered significant if transcript levels between test (NMU) and control (mock) group were differential with a minimal change in expression by 1.5-fold and a raw P value < 0.05. Genes depicted in a hatched fashion did not meet the filtering parameters. Potential crosstalk between WNT and other signaling cascades modulated by NMU over-expression is indicated by dashed arrows.

Supplementary File 6: TCGA breast cancer sample ID

See Supplementary File 6

Supplementary File 7: Primer sequences and PCR conditions for RNA expression analyses

Primer	Sequence	Product size [bp]
***CD44* forward**	5’- GCATCGGATTTGAGACCTGC -3’	125
***CD44* reverse**	5’- GGAGGTGTTGGATGTGAGGA -3’	
***MYC* forward**	5’- ATTCTCTGCTCTCCTCGACG -3’	155
***MYC* reverse**	5’- AGCCTGCCTCTTTTCCACA -3’	
***DKK1* forward**	5’- CCCCGGGAATTACTGCAAAA -3’	142
***DKK1* reverse**	5’- AGACAAGGTGGTTCTTCTGGA -3’	
***FZD3* forward**	5’-CTTTGTGCACTCTACGCTCC-3’	123
***FZD3* reverse**	5’-GGCCAAGGAACACCAAACAT-3’	
***GAPDH* forward**	5’-GAAGGTGAAGGTCGGAGTCA-3’	289
***GAPDH* reverse**	5’-TGGACTCCACGACGTACTCA-3’	
***GHSR1a* forward**	5’- ACCAGAACCACAAGCAAACC -3’	141
***GHSR1a* reverse**	5’- GGCTGATCTGAGCAATCTCC -3’	
***GHSR1b* forward**	5’- CTTGGGACACCAACGAGTG -3’	263
***GHSR1b* reverse**	5’- AGGACCCGCGAGAGAAAGC -3’	
***LRP5* forward**	5‘-AAGCTGTGAATGTGGCCAAG-3‘	152
***LRP5* reverse**	5’-CACGATGCAGGTCTTCATGT-3‘	
***LRP6* forward**	5‘-GCATGTGATTGGCTTGGAGA-3‘	176
***LRP6* reverse**	5’-TCTCCCCAGTCTGTCCAGTA-3‘	
***NMU* forward**	5’- GGATTACAGCCTGAACAACAGC -3’	143
***NMU* reverse**	5’- GGCTTTGGTAGCATTCCCATA -3’	
***NMUR1* forward**	5’- CAGCCAGGTCCAGATACACC -3’	164
***NMUR1* reverse**	5’- CAGGCCATCTGTCCACTGT -3’	
***NMUR2* forward**	5’- CATCATCCAGGTCACCTCCT -3’	120
***NMUR2* reverse**	5’ TTCCCTTCATCTGCCTCAAG -3’	
***NTSR1* forward**	5’- ACCGTCAAGGTCGTCATACA -3’	170
***NTSR1* reverse**	5’- ATGCTGAATGTGCTGTGCTC -3’	
***ROR1* forward**	5’-AGCCATACAGAGGGATTGCA-3’	167
***ROR1* reverse**	5’-GGAAGGAATGGCGAACTGAG-3’	
***WNT11* forward**	5’- CGTGTGCTATGGCATCAAGT -3’	144
***WNT11* reverse**	5’- GTGTGCATGAGCTCCAGGT -3’	

Real-time PCR reaction volumes of 20 µl consisted of the following components:
5 µM forward primer, 5 µM reverse primer, 10 µl SYBR GRN Supermix and 1 µl of cDNA as PCR template. Cycle conditions: 95°C for 3 min, 40 cycles of 95°C for 30 s, 60°C for 20 s, 72°C for 30 s. bp: base pairs.
